# Personalized Medicine, Storied Past, Contentious Present, Promising Future

**DOI:** 10.3390/jpm16040217

**Published:** 2026-04-16

**Authors:** Kenneth P. H. Pritzker, Arash Samari

**Affiliations:** 1 Department of Laboratory Medicine and Pathobiology, Temerty Faculty of Medicine, University of Toronto, Toronto, ON M5S 1A1, Canada; 2 KeyIntel Medical Inc., Toronto, ON M5V 1R7, Canada; asamari@keyintelmedical.com

**Keywords:** Personalized Medicine, Evidence-based Medicine, precision medicine, Stratified Medicine, individualized healthcare, data quality, history of medicine, artificial intelligence, pharmacogenomics, medical informatics

## Abstract

Personalized Medicine has been a central aspiration of medical practice and has guided the direction of medical advances from ancient times to the present. This narrative review highlights some of the most significant past advances and present practices, discusses issues currently limiting Personalized Medicine and proposes activities necessary for Personalized Medicine to have a promising future. Throughout history, Personalized Medicine has developed along with the evolution of science and societal concepts. Notable advances paralleled the growth in what an individual person is and how experimental science can apply to medical practice. In the twentieth century, the study of inborn errors of metabolism and pharmacogenetics broadened the horizons of what Personalized Medicine could be. Presently, Personalized Medicine is challenged by different perspectives on its scope, by the various clinical scientific activities which can inadvertently or by misinterpretation serve to depersonalize medicine, and by the difficulties involved in integrating the massive amount of available scientific data to optimize medical practice centered on the individual. The conditions necessary for Personalized Medicine to have a promising future include developing broader, deeper, and more dynamic knowledge of disease processes, new methods to identify anomalous, singular disease-contributing characteristics in individuals, and improving data quality in research and medical practice. Advancing Personalized Medicine requires developing new perspectives for research, healthcare education, medical practice, and healthcare governance, as well as deploying medical advances at scale across populations.

## 1. Introduction: What Is Personalized Medicine?

Personalized Medicine can be defined as medical practice centered on medical art and the individual person, optimizing both art and science, to address the prevention, diagnosis and treatment of particular diseases, while understanding and respecting the individual’s particular social, cultural physiologic and psychologic condition.

This comprehensive definition, focused on the individual person, has been the essence of medical practice reaching back at least 5000 years, and remains intact today, with growing momentum. This narrative review will consider Personalized Medicine’s storied past, its contentious present, and its very promising future.

Our intent is to show that while the details of medical art and science continue to evolve, the principles of Personalized Medicine endure. Given favorable conditions, the power of Personalized Medicine as a beneficial societal construct will continue to grow at an accelerating rate.

## 2. Personalized Medicine: Storied Past

The history of Personalized Medicine extending back to deep antiquity is vast, necessitating only a few brief sketches to illustrate its growth and evolution. Commonly, the birth of Personalized Medicine refers back to the times of Hippocrates, and the Greek lands in and around the Aegean sea, 2500 years ago [1,2]. In this setting, to control and cure diseases, the emphasis in medicine was directed towards providing the individual with a salubrious climate, good nutrition, hygiene, judicious application of herbal remedies, and attention to mental health. Indeed, medicine in all ancient civilizations, including China, India, Persia, and Egypt, focused on the diseased individual, and brought all the applicable concepts in art and science within each particular civilization to medical practice [3].

Perhaps the oldest recorded specific example of Personalized Medicine was the admonition of Pythagoras, 510 BC, in Croton, Magna Graecia (South Italy), to not eat beans because of adverse reactions in some individuals and families [4]. We now know that this refers to not eating fava beans, as ingestion in susceptible people can sometimes induce fatal hemolytic anemia related to genetic glucose-6-phosphate dehydrogenase deficiency [5].

By 150 AD, exemplified by Galen of Pergamon, careful clinical observation on select populations, such as wound healing in gladiators, spoke to disease classification and the rise in specialized individual practice [6]. Further, Galen and other physicians of his time traveled widely to learn the best medical practices, which were then taught to the generations of physicians that followed.

With the Renaissance starting circa 1500 AD, there was a great expansion of medical knowledge, with the development of formal medical schools such as at Padua [7], as well as an expanded concept of the characteristics of an individual person. Notable advances included the alchemist Paracelsus (1493–1541), who taught that, depending on dose, the same substance could be a drug or a poison, contributing to the origins of both pharmacology and toxicology [8]. Extremely influential was the humanist Montaigne (1533–1592), who, through his essays, described what it is to be truly human [9].

As the Enlightenment approached, empirical science overtook broad theoretical systems. Most dramatic was the demonstration of Morgagni (1682–1771), which showed that specific diseases could be distinguished by morphologic appearances in particular organs and tissues [10].

By the beginning of the twentieth century, rapid advances in science provided overconfident, mechanical descriptions of physics, chemistry, and biology, fostering ideas that each natural process was structured as a continuum that could be interrogated and manipulated as a group. These ideas, together with successful population-based medical advances against disease, such as hygiene, water purification, vaccination, and nutrition (vitamins), diminished the emphasis on Personalized Medicine. Against this background milieu, a pediatrician, Archibald E. Garrod, by carefully observing physical signs and by making careful biochemical analyses of urine, recognized that some rare diseases, such as alkaptonuria, termed inborn errors of metabolism, were associated with human individual variation within families [11,12]. While Garrod’s specific medical advances were widely recognized, his most far-reaching idea, that some diseases were associated with intrinsic individual variation, was not. This lack of recognition can be ascribed in part to the contrary evidence that population-based interventions could reduce the prevalence and severity of diseases and in part to the fact that genetic science applied to medicine was in its infancy. Genes were yet to be associated with human variation or with specific diseases [13].

**Figure 1:**
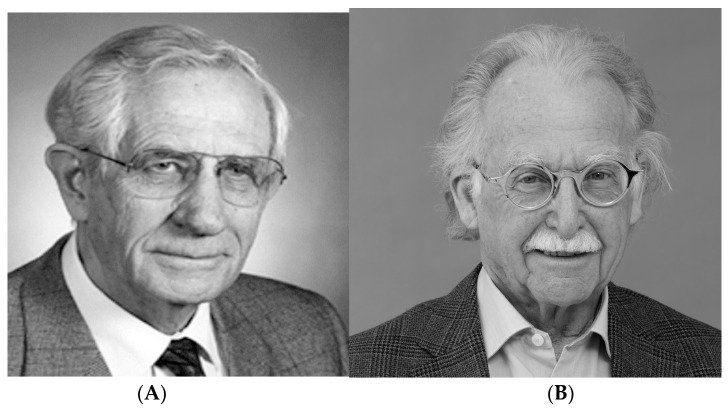
(**A**) Werner Kalow, Courtesy, Malignant Hyperthermia Association of the United States; (**B**) Urs Meyer, licensed under CC BY-SA 4.0, via WikimediaWikipedia.

By the 1950s, medical science was becoming focused on why some uncommon medical conditions occurred in particular individuals. Of particular importance for the development of Personalized Medicine was Werner Kalow’s biochemical studies of individuals experiencing adverse reactions to local and systemic anesthetic agents [14,15] (Figure 1A). By examining the blood of affected individuals and healthy controls, Kalow demonstrated that affected patients had variants in cholinesterase activity, which made them and some members of their families much more sensitive to these drugs [16,17]. In the early 1960s, as a very young summer student in Pharmacology at the University of Toronto, I was aware of Prof. Kalow and his pioneering work on Pharmacogenetics. Only retrospectively did I appreciate the singular significance of Pharmacogenetics for Personalized Medicine and how much can be learned by careful study using simple, relatively inexpensive techniques such as electrophoresis and spectrophotometry to assess enzyme structural differences and enzyme activity. By the end of the twentieth century, with advances in understanding gene structure and function, Pharmacogenetics expanded from genetic mutations affecting drug responses to encompass drug-induced genomic activity enhancement and inhibition [18]. Pharmacogenomics has emerged as a major discipline not only for adverse drug reactions but also for understanding gene–drug–drug interactions [19,20]. To this discipline and its clinical application, Prof. Dr. Urs Meyer, the first Editor of the Journal of Personalized Medicine, was a notable contributor, particularly in the application of gene polymorphisms to drug metabolism and drug–drug interactions [21] (Figure 1B).

## 3. Personalized Medicine: Contentious Present

**Figure 2:**
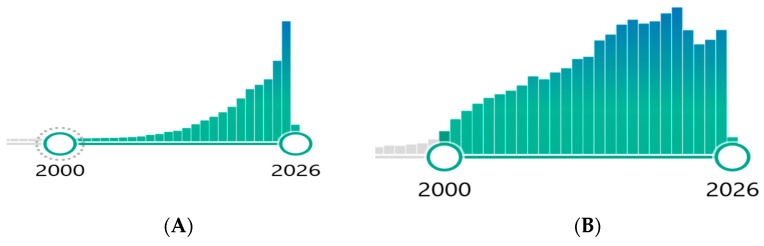
Publications Timeline 2000–2026. (**A**): Personalized Medicine, (**B**): Pharmacogenomics. PubMed: (**A**) Personalized Medicine: https://pubmed.ncbi.nlm.nih.gov/?term=personalized%20medicine&sort=date&timeline=expanded (accessed on 1 February 2026); (**B**) Pharmacogenomics: https://pubmed.ncbi.nlm.nih.gov/?term=pharmacogenomics&filter=years.2000-2026&timeline=expanded&sort=date (accessed on 1 February 2026).

Personalized Medicine as an investigative concept, is in a rapid growth phase. A survey of PubMed publications shows that papers with the term “Personalized Medicine” tracking about 500/year prior to year 2000, has now reached 5000/year in 2026. A similar growth pattern is seen for “Pharmacogenomics” [22] in Figure 2.

However, against total biomedical publications/year > 4,000,000, Personalized Medicine appears as a very small, specialized subject. From the 1960s to the present, there have been vast advances in biological science and its application to Personalized Medicine. Four highlights, discussed below, are selected as examples of the current Personalized Medicine practice frontier. These advances are clinical pharmacogenomics applied to drug selection and dosage, hypersensitive adverse drug reactions, HLA system associations with individual immune system dysregulation, multi-omics as Personalized Medicine diagnostic tools, and gene editing therapy for rare diseases.

Pharmacogenomics, through advances in pharmacokinetics and pharmacodynamics, has expanded Personalized Medicine applications from detection of adverse drug reactions to include a broader understanding of gene–drug–drug interactions. This has increased drug efficacy, tolerability and safety in individuals by appropriate drug and dose selection and by identifying gene variants that can enable novel drugs for targeted therapy [18,23,24,25,26]. In affected persons, gene variants can increase or decrease drug activity, availability and drug–drug interactions by producing protein variants that affect the function of critical cell components such as drug metabolizing enzymes, receptors, or transport channels [27]. Relevant genes, “pharmacogenes”, include genes involved in drug transport, drug metabolism, and immune reactions to drugs. Particularly notable has been the detection of cytochrome 450 oxidoreductase enzyme allelic variants involved in drug metabolism [23,24,28]. Beginning with the Roche Amplichip, circa 2005, functional genotyping methods for drug-metabolizing enzymes have been utilized in clinical practice [25,29]. Presently, multigene panels are available that can be used prior to drug therapy to prevent adverse drug reactions [26]. Important for Personalized Medicine is phenoconversion, as seen with the CYP2D6 phenotype, whereby the pharmacogenic effect on drugs as widely varied as opioids (codeine, tramadol), tricyclic antidepressants (e.g., amitriptyline), and estrogen receptor modulators (e.g., Tamoxifen) are modified or annulled by real-world non-genetic factors [30]. Clinical practices in pharmacogenomics are actively fostered through the international activities of the Clinical Pharmacogenetics Implementation Consortium (CPIC), which provides continually updated educational resources [31].

Human Leukocyte antigen (HLA) proteins on cell surfaces generated by genes on chromosome 6 are the primary regulators of tissue histocompatibility and immune responses. More than 15,000 allelic variants have been identified, with distinct variants associated with predisposition to immune dysregulation or chronic inflammatory diseases ranging from inflammatory bowel disease to axial spondyloarthritis [32,33,34,35,36]. As examples, important actionable HLA gene variants for cutaneous drug hypersensitivity reactions in specific populations include HLA-B*57:01–abacavir in Caucasians, HLA-B*15:02, Carbamazepine in Taiwanese, Chinese, and HLA-A*11:0 1 Salazosulfapyridine in Japanese people [35].

Multi-omics is the collective term for massively parallel molecular analysis of genes, ribonucleic acid transcripts, proteins, and post-translational protein fragments [37]. Integrated multi-omic analysis is starting to provide insight into individual variation for many different diseases [38,39], such as diabetes mellitus [40], osteoporosis [41], inflammatory bowel disease [42], kidney stones [43], gout [44], and some cancers [45].

Rare diseases are disorders that affect <0.1% of the population [46]. The chronicity, severity and molecular heterogeneity of rare diseases have stimulated many advances in multi-omic approaches to diagnosis for diseases, such as birth defects [47] and rare genetic diseases [48]. This has led to novel individualized therapy, including gene editing for particular diseases [49]. Novel clinical approaches to individuals with these diseases are providing leading examples of Personalized Medicine implementation.

Even with steady clinical implementation progress, Personalized Medicine, at present, is a contentious subject. There are three major threads: Personalized Medicine scope, formal clinical research methods, which unintentionally pay less attention to individual variation, thereby tending to depersonalize medicine, and public societal influences, which tend to promote the term “Personalized Medicine” widely. First, with regard to scope, there is a wide spectrum of what might be termed Personalized Medicine, ranging from a narrow definition concerned with a more personal medical approach to an individual with a disease to the broadest definition, which includes measures to foster wellness, longevity, as well as physical and mental performance enhancement.

However, as currently understood, the main determinants of human health, which include adequate nutrition, an environment free of pollutants, education, employment opportunity, and social cohesion, are societal factors independent of medical practice [50]. Yet these determinants do weigh on the presence, type, and severity of disease in the individual as well as treatment responses.

Second, the forces which may inadvertently *depersonalize* medicine are pervasive, as seen by large-scale but fragmented ‘omics’ science [51] and informatics using pattern-seeking artificial intelligence, large language models [52,53], without regard to biological hypothesis or focus on individual variation. Third, the term “Personalized Medicine” is attractive to the public consumer. Accordingly, “Personalized Medicine” is used widely to promote various commercial health-related products, whether or not these products are truly focused on the characteristics of the individual.

As a cornerstone of practice, contemporary clinical medicine utilizes extensive knowledge developed from Evidence-Based Medicine [54], Stratified Medicine [55], and associated systematic reviews [56].

Evidence-Based Medicine is the practice of medicine, based on the best scientific evidence integrated with the collective experience of the physician, together with the condition and wishes of the patient [57]. Stratified Medicine involves a strategy to utilize specific biomarkers to determine the likelihood of the patient responding to a specific treatment [55].

These methods have advanced Personalized Medicine by identifying optimal therapy for selected patient groups and by recognizing side effects peculiar to individuals that share similar disease characteristics. Laudable and accomplished as these methods have been to advance medical practice, too often individual variation is underestimated, thereby becoming inadvertently suppressed. While effective for their intended purposes, these practices can at times be unintentionally adverse for Personalized Medicine. List 1.

**List 1.** Some practices that may contribute inadvertently to *depersonalize* medicine.
Evidence-Based MedicineStratified MedicineComparative effectiveness researchConventional statisticsPopulation health studiesPrecision medicine (hitting a target closely but missing the most relevant target)Clinical GuidelinesBroadening Drug LabelsReductionism, Fragmented ScienceArtificial intelligence marketing hypeAutomated Medicine


Evidence-Based Medicine research is frequently concerned with comparing relatively large patient cohorts to determine relatively small differences in therapeutic efficacy, quite frequently between different drugs of the same class. In this process, statistical techniques are used to suppress or discard outliers, individuals that do not fit in some characteristics with the bulk of the cohort.

Comparative Effectiveness Research seeks to frame the benefits and harms of different diagnostics, treatments and preventative practices in typical patients to define the most effective and most efficient interventions. This research is highly dependent on biomarkers with firmly rooted biologic rationale as well as excellent test performance and reproducibility characteristics. Of necessity, Comparative Effectiveness Research considers groups of “typical patients” thereby potentially obscuring best outcomes for individuals whose disease presentation or treatment response is atypical [58,59,60].

Precision medicine is an evolving term that currently refers to diagnostic multi-omics biomarker methods that utilize knowledge gained in genomics, transcriptomics, proteomics, metabolomics, epigenomics, and microbiomics to identify patient-specific therapies [61]. Precision medicine, most frequently employed in oncology, utilizes knowledge of genomic variants in large cohorts to predict therapeutic susceptibility to agents highly targeted against a specific genomic mechanism [62,63]. Whether precision medicine can be truly personal at present is debatable [64]. Related to its current biomarker imprecision [65], at best, the concept of precision medicine as Personalized Medicine must be considered aspirational. At present, precision oncology is more useful for determining lack of susceptibility rather than effectiveness and ignores ineffective results that may result from the biologic process that bypasses the drug target in individuals. Clinical Guidelines reflect current accumulated knowledge and broad committee consensus [66], both imperfect, again paying insufficient attention to individual variation.

In the past, pharmaceutical corporations were interested in broadening drug labels to affect larger populations, thereby increasing the market for each drug [67]. Recently, this has been changing as drug approval and reimbursement by health payors is becoming dependent on drug efficacy. Both broadening and narrowing drug labels can be detrimental to identifying individuals who can or cannot respond unless there are pharmacogenomic or other specific tests available to guide individual responsiveness. As noted above, reductionism and fragmentation of sciences can falsely promote very particular molecular biological mechanisms for therapeutic targeting, whereas treatment effectiveness may be dependent on the individual’s broader integrative capacity to respond.

Population health is concerned with health system strategies to decrease the incidence or severity of diseases [68]. The techniques used may not prevent disease in some individuals most susceptible and may be redundant in individuals most resistant to disease. In both instances, individuals may have increased susceptibility to treatment side effects. Not least, the current fashionable hype about artificial intelligence, with regard to consumer disease diagnosis and treatment, ignores human professional expertise focused on the individual’s condition [69].

The sheer immensity of integrating large amounts of data with dynamic developments in biological mechanisms and emerging understanding of the individual human condition represents formidable challenges to Personalized Medicine implementation [70].

In contrast, Personalized Medicine is focused on assessing individual differences, as shown in List 2.

**List 2.** Personalized Medicine: Characteristic Features.
Individual variation in disease status as elicited by clinical history and physical examination.Individual biological variation at multiple scale levels, including gene polymorphism, RNA splicing, protein polymorphisms, and post-translational protein modifications.Individual heterogeneity in sporadic diseases and rare diseases.Individual environmental history, geographical location, and social circumstance.Individual psychological condition and cultural outlook.


Crucial to understanding the essence of Personalized Medicine is the first encounter between physician and patient, early in the disease. At this time, both the nature of the disease and the patient’s specific condition are unknown until elicited by the physician’s skills in obtaining a detailed history and performing a physical examination. Typically, further assessment is required involving imaging, laboratory, and perhaps physiological tests. Usually, after a history and physical examination, the disease state at presentation becomes defined and appropriate treatment is started. However, with chronic diseases, further physician–patient encounters and further tests may be required to appreciate the patient’s disease status and how best to approach the patient’s treatment. Even then, in some patients, elements of active disease may remain ambiguous; patients with the same disease may display different symptoms and signs; and patients with different diseases may display similar symptoms and signs. A consequence is that recorded data used for scientific study may contain many uncertainties, potentially confounding interpretations by every data analysis technique, including those of artificial intelligence.

The preceding 50 years have revealed enormous knowledge at different levels of human biological organization, illuminating structures and functions of genes, RNA transcripts, proteins and the deep complexity of anabolic and catabolic processes. A core idea for Personalized Medicine is to integrate this knowledge with correlated observations in individuals, across diseases and populations, to obtain comprehensive linked databases that can be used for individual patient assessment [71]. However, at a molecular level, the sum of genomic, transcriptomic, proteomic, and post-proteomic molecular variations identified to date, their permutations and combinations are so great as to defy complete analysis by the most powerful computers [72]. Just in the discipline of pharmacogenomics alone, future developments envisage integrating elements of epigenetics, nutrigenomics, microbiomes, protein interactions, exosomes, and metabolomics [73]. Further obstacles include difficulties in obtaining data, data quality and the prohibitive costs of this strategy [74].

Yet, at the practical level, these advances have led to approaches to personalized diagnosis and therapy through techniques applied in prenatal screening, neonatal screening, pharmacogenomics, precision oncology and rare diseases.

To determine in advance the best drug for an individual, companion diagnostics using molecular biochemical tests of blood or tissues have been applied widely. To date, these tests have been more useful in determining which patients are unlikely to respond than in selecting patients who will have the most effective responses.

The very substantial advances in understanding molecular biochemical processes have led to the identification of biochemical motifs as drug targets and drug development to address drug targets as blockers or agonists. As noted above, companion diagnostics have been devised to determine whether individuals will respond to drugs addressed to specific targets. While substantial progress is being made and individual patients’ success is encouraging, it is still common for patients who appear susceptible to targeted therapy to have disease progression.

Amongst the major issues confronting Personalized Medicine implementation, high operational costs and dubious data quality stand out as barriers. Widespread Personalized Medicine implementation requires extensive “omic” diagnostics and informatics, which are perceived as prohibitively expensive to apply at the population scale. However, the costs of both activities are dropping rapidly. With the Genome Wide Association Study (GWAS) and other technologies, whole genome analysis with reduced computational burden is now approaching the analytic cost of $100 USD [75]. The current huge general investments in artificial intelligence computing and networking are enablers of the informatics infrastructure required for Personalized Medicine. To provide Personalized Medicine at scale will require augmented and continuing investment in electronic communication and education technology so that non-Personalized Medicine expert healthcare practitioners can have access to the domain knowledge and consensus clinical guidance. Particularly important for diagnostics will be practical guidance, decision support on health/disease analyte thresholds and clinically relevant grades of elevated or low analyte concentration.

Diagnostics represent only about 5% of direct healthcare costs [76]. If there were a strategy to shift 1–2% of healthcare costs to diagnostics, this could save 10–20% of healthcare treatment costs and achieve better healthcare outcomes, together with immense population and economic benefits. If better healthcare outcomes at lower cost are an agreed societal goal, this strategy is feasible and practical on an ongoing incremental basis. What is required is a more complete collection of better patient care data, better analysis of data collected, more effective communication of knowledge gained, and, most importantly, societal will. Contemporary informatics solutions [77] and the networked computational storage, analysis and distribution power, are being established. Current obstacles to implementation of this strategy include fragmented privacy legislation and lack of political priority to reform healthcare.

Regarding data quality, examples of problems include the healthy reference range for blood analytes and the application of synthetic data derived from artificial intelligence models. The assessment of “normal” for healthy individuals’ blood analytes [78], and by extension, the reference range with cutoffs between normal and abnormal (elevated or depressed) is a critical, unsettled subject [79]. While this problem is most apparent for blood analytes for which population and individual data are abundantly available both for healthy individuals and those with disease, arguably, this concern applies to all phenotypic characteristics that have parametric dimensions. This issue came about because, at the end of the 19th century, human data variation was considered to follow a “normal distribution”. A normal distribution, as defined by Gauss, is a smooth distribution centered on a mean value [80]. Commonly, for blood analytes, normal distribution is defined as the mean ± 2 standard deviations. For Personalized Medicine, there are three major problems with this concept. First, from genetics, it is known that some individuals who have levels outside the recognized normal range can be healthy and that some individuals with analytes inside the normal range can have an abnormal value for the individual because of genetic or acquired insensitivity to the analyte level. For both statistical outliers and inliers, distinguishing between truly anomalous data and biological “noise” can be truly challenging. The signal for further investigation in these situations resides in expert domain expertise that can compare the collective observed data to the lack of fit with the patient’s condition [81].

Second, analysis of multiple common blood analytes in healthy individuals shows that the distribution of each analyte is different, with some analytes showing a positive or negative skew distribution, and others, discrete, narrow or broader clusters. Third, the population sampled is an issue. Typically, a known healthy population of a particular age range, male and female, is selected, with many exclusions related to ambient diseases, medication history, etc. This selection assumes a sharp demarcation between healthy and diseased individuals. A broader population approach may show that an analyte presence or concentration may have considerable overlap between healthy and diseased populations, indicating that some healthy individuals can have transient elevations which, although outside the “normal range”, remain healthy. These considerations point to the concept that healthy features can encompass a broader distribution than the normal range and that, for some individuals, an analyte within the normal range can represent disease. If all individuals, healthy and diseased, are considered together, the healthy/disease cut-off for increase and decrease for any given blood analyte may appear to be arbitrary. Therefore, the clinical utility of the observed increase or decrease in an analyte is highly context-dependent on the individual, the disease and the patient’s condition.

At present, throughout the world, scientists and laypersons as media consumers are bombarded by promises touted by artificial intelligence companies that individualized medicine is almost here, available to everyone (for a price) through artificial intelligence techniques utilizing massive amounts of data analyzed through large language models [82,83]. The availability of large datasets is usually restricted to population health studies and very large clinical trials. Unsupervised Learning applied to large language models with massive datasets can be very useful to demonstrate formerly unlabeled patterns with many medical applications, including the discovery of novel disease subtypes, biomarkers, medical image analysis, and patient stratifications [84,85]. Yet, substantial conceptual, reproducibility and safety difficulties with this approach need to be recognized [82,86,87]. The first problem is data integrity. With every observational and experimental study, there is some uncertainty about each data point. As data is amassed, the uncertainties increase and validating conclusions becomes more complicated. Second, large datasets can reduce random noise from insufficient data but at the risk of amplifying hidden bias. The more data that is analyzed, the greater the probability that patterns will be discerned. At the same time, the larger the dataset, the less likely that the patterns can be replicated. Third, replicating very large datasets of human diseases is both impractical and so costly that rigorous validation may not be possible. Nonetheless, partial validation may be possible and, if done well, can contribute to the ladder of evidence for the pattern discovered.

Artificial intelligence safety risks are multitudinous. Amongst these risks, false synthetic data, “hallucinations’, and risks to individual privacy are of paramount concern [88,89]. Advances in communication and education technology are required to mitigate these risks and ensure patient safety.

Clinical studies based on disease cohorts present in a single institution or an uncommon disease process can be amenable to artificial intelligence/machine learning techniques. These techniques can be of considerable value to Personalized Medicine when limited datasets of closely related parametric and non-parametric (binary) information are applied to individual patients, informed by extensive domain knowledge [90,91,92,93,94,95]. These techniques, too, have their limitations, of which overfitting synthetic data leading to unsupported conclusions, is amongst the most prominent [96,97,98].

Current artificial intelligence medical applications include tools to facilitate better notetaking, techniques to improve medical education and training, as well as resources to assist medical diagnostics and treatment [90]. Domain knowledge can restrict what data is allowed to enter analysis and shape the analysis to address the most clinically relevant questions with outputs designed for clarity of clinical interpretation. Utilizing domain knowledge, informative data can be analyzed in an effective step-wise, orderly process. Key questions are framed prior to analysis, including what domain experts, such as specialist physicians, would find useful to guide assessment of individual patients, and how best to categorize the assessment. Using chronic inflammatory diseases as an example, data can be collected that is likely to be most informative; data can be analyzed to distinguish diseases by different inflammation patterns, and the sensitivity and specificity for the analyte patterns can be characterized. If inflammation is present, the inflammation intensity can be assessed in grade categories that clinicians understand, and these methods can be applied to individuals across populations serviced by different labs.

Assembling data, whether this is in single observations of symptoms, physical signs, imaging, or lab tests or whether the observations are an assemblage of massive data analyzed by machine learning or whether it is set of recommended actions with probabilities generated by the sum of related experience sorted within a computer, is only an initial step in Personalized Medicine. The next step is for the physician to evaluate the data and then, together with the patient’s informed consent, to initiate a treatment plan that would be monitored for effectiveness and modified as necessary over time. To be most useful, the biomarkers must be highly selected, readily accessible, and obtained in time intervals pertinent to the patients’ condition.

## 4. Personalized Medicine: A Conditional, Promising Future

From ancient times to the present, the pursuit of Personalized Medicine is recognized as a most worthy societal aspiration. Throughout history, Personalized Medicine has been based on both the available biological science knowledge as well as the wisdom conferred by deep learning in the humanities applied to the individual, their particular diseases and social circumstances. The difficulties with approaching medical practice from a Personalized Medicine perspective have been reviewed in the context of the COVID-19 pandemic [99]. Current limitations in applying Personalized Medicine include inadequate understanding of disease pathogenesis thereby affecting adversely, current therapy; inadequate knowledge of synergistic and antagonistic gene–drug–drug interactions; and the inadequate application of sensitivity vs specificity in diagnostic test development [99].

To achieve the goals of Personalized Medicine for broad populations, new perspectives on developing and applying our knowledge will need to be constructed. First, it will be necessary to develop broader, deeper, and more dynamic knowledge of individual humans, their changes over time, as well as of their growth, aging and diseases (predisposition, preclinical onset, pathophysiology, natural history, treatment effects and side effects, and outcomes). Examples of this approach include, in basic science, the spatial and temporal dynamics of tissue inflammation and repair [100], as well, at the clinical practice level, the effect of individual circadian rhythms on disease activity and drug responses [101].

Second, to advance Personalized Medicine, there is a need to develop methods to identify in the individual, unexpected disease and therapeutic response features that are anomalous and that may be singular or an outlier [102]. The development of point-of-care molecular diagnostics devices will be useful tools for timely Personalized Medicine diagnosis [103]. Third, special attention will be needed to improve data quality and verification techniques to ensure that data quality is, in fact, high. “New” mathematical techniques for multidimensional analysis beyond conventional population statistics will be needed to demonstrate the relative relationships of different data types to each other. Further, data analysis will need increased focus on addressing questions of high clinical utility using novel informatics methods, including unsupervised machine learning informed by domain expertise.

Regarding health systems, to achieve the data assembly goals, large health systems will need to be linked to build very large, very deep databases. As important as data standardization amongst health systems is the challenge to bring together, in an orderly and effective way, the massive amounts of data, which faces numerous obstacles related both to preserving individual privacy and to obtaining capital and maintenance funding. Accordingly, the solutions may reside in completely alternative approaches using selected key-related data to quickly ascertain the disease state of individuals. This approach is within sight for chronic inflammatory diseases and likely not that far removed for neoplasia. As these capacities are developed for these disease classes, the methods can be adapted for others, perhaps with priorities to include pandemic infections and chronic degenerative disorders.

## 5. A Personalized Prescription for Personalized Mediciner

The future of Personalized Medicine is promising, but the promise is conditional on the wise application of resources which draw on our emerging knowledge of biology, social sciences, informatics, computer structures, health systems and their management.

To achieve the most promising future for Personalized Medicine, specific activities in research, education, medical practice and healthcare organization are recommended. For research, priority should include finding better methods to improve and verify data quality [104,105]. This includes developing a better understanding of the limitations of multivariate analysis, as well as understanding and developing the use of closely related data as probes for specific disease process patterns. Next, medical and more broadly healthcare domain expertise needs to be channeled into clinically meaningful questions, where clinically quantizable activity or severity, as well as outcomes, can be addressed. For education, Personalized Medicine values and envisaged best outcomes need to be reinforced at all levels of healthcare education. In parallel, it is necessary to recruit investigators, healthcare practitioners, and healthcare administrators to become knowledgeable about Personalized Medicine and to become aware of the counterproductive forces inherent in the many activities that may serve inadvertently or by misinterpretation to depersonalize medicine. Ultimately, Personalized Medicine as a goal, value, and practice needs to be adopted widely and deeply into healthcare systems.

List 3 provides examples of current Personalized Medicine practices.

**List 3.** Personalized Medicine Practices, Examples.
Diagnostic methods to assess the anomalous disease presentation, the individual with rare or singular disease, and whether the outlier in individual patient data is important for assessment or is just biological “noise”.Expansion of diagnostic methods to assess pathologic individual genetic variation in screening and diagnostic programs, such as prenatal genetic screening, neonatal genetic screening, rare genetic disease detection, and pharmacogenomics.Treatment methods that narrow drug labels to enhance therapeutic efficacy and minimize side effects.Cost-effective strategies to implement Personalized Medicine. The objective is to devise strategies to achieve better healthcare outcomes while lowering healthcare costs by utilizing better diagnostic methods that result in more effective therapy.


Adopting Personalized Medicine practices is the challenge and responsibility not only of physicians and all other health practitioners but also of healthcare planners, administrators, and healthcare governance officials.

## Data Availability

No new data were created or analyzed in this study. Data sharing is not applicable to this article.
